# The relationship between the level of NMLR on admission and the prognosis of patients after cardiopulmonary resuscitation: a retrospective observational study

**DOI:** 10.1186/s40001-023-01407-w

**Published:** 2023-10-11

**Authors:** Qingting Lin, Nan Zhang, Huadong Zhu

**Affiliations:** Emergency Department, State Key Laboratory of Complex Severe and Rare Diseases, Peking Union Medical College Hospital, Chinese Academy of Medical Science and Peking Union Medical College, Beijing, 100730 China

**Keywords:** Cardiac arrest, NMLR [(neutrophil + monocyte) to lymphocyte], Prognosis

## Abstract

**Background:**

The inflammatory immune response is involved in the pathophysiology of the post-cardiac arrest syndrome and leads to high mortality. The admission (neutrophil + monocyte) to lymphocyte ratio (NMLR) can help us to assess the immune inflammatory status of patients. We aimed to identify factors that affect the prognosis and explore the association between NMLR and the prognosis of patients after cardiopulmonary resuscitation (CPR).

**Methods:**

This is a retrospective study based on the MIMIC-IV database. We assessed patients admitted to the ICU after cardiopulmonary resuscitation, included demographic characteristics, peripheral blood cell count and blood gas indicators for the first time after admission to the ICU, developed a multivariate COX proportional-hazards model to explore prognostic factors, and divided patients into High NMLR and Low NMLR groups by cutoff values of NMLR. Propensity score matching (PSM) was used to adjust confounding factors.

**Results:**

A total of 955 patients were included in the analysis, with 497 surviving and 458 dying during the follow-up period. In a multivariate Cox proportional-hazards model, age (RR 1.007, *p* = 0.0411), NMLR levels (RR 1.003, *p* = 0.0381), lactate (RR 1.097, *p* < 0.001) and hematocrit (RR 1.101, *p* < 0.001) were independent risk factors for patient death following CPR. Patients were divided into a high NMLR group (> 14.2) and a low NMLR group (≤ 14.2) based on the optimal threshold for NMLR. Compared to low NMLR group, high NMLR group had higher total vasoactive drugs and lower 28-day survival. After PSM, there were no differences in baseline characteristics. The high NMLR group still had a higher mortality rate (*p* = 0.001), lower 28-day survival (*p* = 0.001) and shorter length of stay (*p* = 0.005) compared to the low NMLR group.

**Conclusions:**

Age, NMLR levels, lactate levels and hematocrit were independent risk factors for death in patients after CPR. NMLR > 14.2 was associated with higher mortality and was a potential predictor of clinical outcome in patients after CPR.

**Supplementary Information:**

The online version contains supplementary material available at 10.1186/s40001-023-01407-w.

## Background

Worldwide, the incidence of cardiac arrest is high. By continuously improving the early survival chain of cardiac arrest, including early recognition of cardiac arrest, early provision of cardiopulmonary resuscitation (CPR) and early defibrillation, sustained autonomic circulation (ROSC) are better achieved. However, during cardiac arrest and CPR, various organs throughout the body experience ischemia/reperfusion (I/R) injury, which can still result in up to 50–70% in-hospital mortality, or severe organ failure [1–3]. Multiple organ dysfunction, including hemodynamic failure, myocardial damage, and nerve damage, occurs several hours (6–24 h) after ROSC, known as post-cardiac arrest syndrome (PCAS). Ischemia–reperfusion injury and high levels of pro- and anti-inflammatory cytokines in the blood are thought to play key roles in the pathophysiology of cardiac arrest syndrome. These inflammatory factors may play an etiological role in organ injury through endothelial dysfunction, vascular leakage, and vasodilation. In previous studies, an increase in circulating leukocytes after cardiac arrest and differences in the levels of different subtypes of leukocytes and altered levels of several cytokines and C-reactive proteins were found to correlate with the severity of PCAS and help predict neurological prognosis and survival after cardiac arrest. For example, elevated neutrophils have a detrimental effect on myocardial ischemia–reperfusion injury[4, 5].Platelet-to-lymphocyte ratio (PLR) and neutrophil-to-lymphocyte ratio (NLR) are predictors of mortality within 30 days in patients with in-hospital cardiac arrest (IHCA) [6]. Neutrophil-to-lymphocyte ratio (NLR) ≥ 6 is associated with mortality and epinephrine application in patients with cardiac arrest. The level of NLR after ROSC contributes to the prediction of neurological prognosis [7]. NMLR is the (neutrophil + monocyte)/lymphocyte ratio, which has been found to be a prognostic indicator of inflammatory and immune-related diseases in previous studies and is gaining attention as an emerging inflammatory and immune indicator [8]. In patients with acute myocardial infarction (AMI), elevated NMLR levels on admission are an independent predictor of increased in-hospital mortality [9].Inflammation and immune mechanisms play an important role in pathophysiological processes after cardiac arrest, but NMLR has not yet been used to predict the prognosis of cardiac arrest patients. The main objective of this study is to retrospectively examine the association between NMLR levels on admission and the prognosis of patients after cardiac arrest.

## Methods

### Database

We conducted a retrospective study to investigate whether elevated NMLR on admission was an independent predictor of poor prognosis in cardiac arrest based on the Medical Information Mart for Intensive Care Database IV (MIMIC-IV version 1.0). The MIMIC-IV database contains clinical data on more than 40,000 patients admitted to the intensive care unit of Beth Israel Deaconess Medical Center in Boston, Massachusetts, USA, between 2008 and 2019.MIMIC database has received ethical approval from the Institutional Review Boards (IRBs) at Beth Israel Deaconess Medical Center (BIDMC) and Massachusetts Institute of Technology (MIT).In this database, no information about the true identity of the patient is included. Therefore, there is no need to obtain informed consent from the patient. We were permitted to extract data from the database by obtaining the certificate of the collaborative institutional training initiative (CITI) (certificate no.:11000241) at the National Institutes of Health.

### Study population

We extracted clinical information from adult patients ≥ 18 years admitted to the ICU for analysis. Patients diagnosed with cardiac arrest according to the diagnosis codes of the International Classification of Diseases (ICDS) 9th and 10th editions (" 4275 ", "I46", "I462", "I468", "I469") met the inclusion criteria. The following exclusion criteria were used for screening: (1) patients aged < 18 years; (2) missing survival outcome data; (3) pregnant and postpartum patients; (4) patients who presented with cardiac arrest after admission to the ICU; and (5) patients without peripheral blood lymphocyte count, monocyte count or neutrophil count.

### Data extraction

We used the PostgreSQL tool to extract the following clinical and laboratory parameters of patients from pgAdmin and Navicat Premium: (1) demographic characteristics: gender, age, BMI, underlying cardiac cause and place of admission; (2) comorbidities: including sepsis, age-adjusted Charlson comorbidibility Index (CCI), myocardial infarction, congestive heart failure, peripheral vascular disease, cerebrovascular disease, dementia, chronic lung disease, rheumatic disease, peptic ulcer disease, liver disease, paraplegia, kidney disease, diabetes, malignant tumors, metastatic solid tumors, acquired immune deficiency syndrome (AIDS); (3) laboratory indicators measured for the first time on admission: leukocytes, neutrophils, lymphocytes, monocytes, platelets, basophils, haemoglobin, haematocrit, eosinophils, pH, lactate, pCO2, PaO2/FiO2, NMLR (neutrophil count + monocyte count)/lymphocyte count; (4) scoring system: sequential organ failure assessment (SOFA) score calculated within 24 h of ICU admission; and (5) treatment: whether mechanical ventilation and defibrillation were performed after admission to the ICU; vasoactive drug dosage.

### Primary and secondary outcomes

The primary outcome was in-hospital mortality. Secondary outcomes included 28-day mortality (28_death), length of ICU stay (los), length of hospital stay, and vasoactive drug dosage (including antidiuretic hormone, dopamine, and norepinephrine) during ICU stay. Patients were divided into survivor and mortality groups according to their primary outcome.

### Statistical analysis

Variables with less than 25% missing values were retained in the data cleaning process. We then used the multiple interpolation method in the MICE R package to estimate the missing values of the selected variables to fill in. Shapiro–Wilk test was used to test the normality of continuous variables. If the variable conforms to the normal distribution, it is expressed by mean ± standard deviation; Otherwise, it is the Median and quartile interval (IQR, p25–p75). Continuous variables were then compared using the student *t* test or Wilcoxon–Mann–Whitney test. Categorical variables are expressed in frequency (percentage) and tested using Chi-square tests or Fisher exact tests. Variables that were statistically different in the univariate analysis were included in multivariate Cox regression proportional hazard model to find factors that influenced patient survival after cardiac arrest. In addition, the best cutoff values for NMLR were obtained using the survminer package and the survivor package. Patients were then divided into two groups based on the optimal cutoff value, the high NMLR group (> 14.2) and the low NMLR group (≤ 14.2). Kaplan–Meier method was used to analyze the relationship between NMLR levels and 28-day mortality. Kaplan–Meier survival curves of the two groups were plotted using survival packs, and differences between the two groups were compared using log-rank test. To reduce the effect of confounding factors, propensity score matching (PSM) was used to balance the baseline characteristics between the high and low NMLR groups to further analyse the relationship between NMLR levels and clinical outcomes of patients after cardiac arrest. ROC curves were used to compare the level of NMLR, NLR, PLR and lactate in predicting in-hospital mortality after cardiopulmonary resuscitation. All statistical analyses were performed using R software. *P* < 0.05 was considered statistically significant.

## Results

**Fig. 1:**
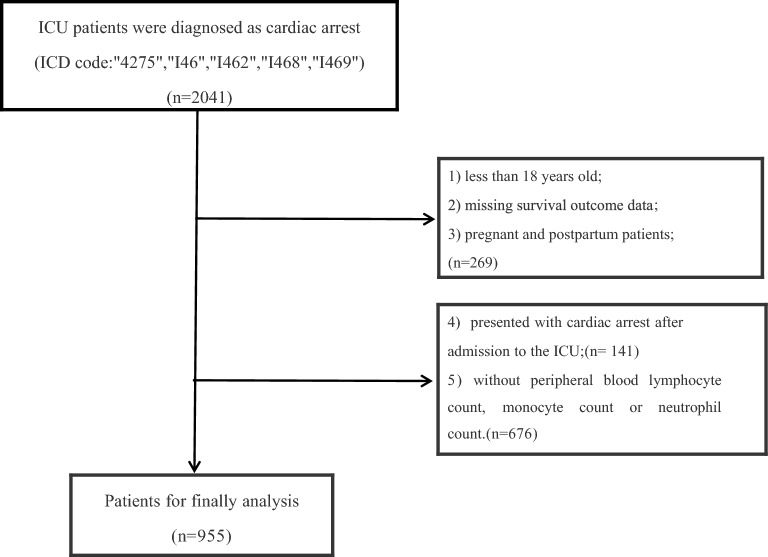
Flowchart. ICU, Intensive Care Unit; ICD, International Classification of Diseases

A total of 1772 ICU patients met the inclusion criteria, excluding patients with missing important clinical indicators (*n* = 676) and patients who suffered cardiac arrest after admission to the ICU (*n* = 141), resulting in a total of 955 patients included in the analysis (Fig. 1). During the follow-up period, of the patients included, 497 survived and 458 died. There were 395 (41.4%) female patients. The median age of the patients was 66 years. 74 (7.7%) of the patients had an underlying cardiac cause. 541 (56.6%) of the patients were admitted via the emergency route. Baseline characteristics of the survivor and mortality groups were compared and statistical differences were found in the distribution of gender, age, BMI, diagnosis, CCI score, NMLR, PaO2/FiO2, ph, lactate, hematocrit, and hemoglobin (Table 1).We found that defibrillation rates were lower in both the death and survival groups. To explore the clinical characteristics of patients who underwent defibrillation, we compared baseline characteristics of patients by grouping them with and without defibrillation, as described in Additional file 3.

**Table 1:** Baseline characteristics of all patients, and of patients grouped by survival outcome

	Overall (*n* = 955)	Survival (*n* = 497)	Death (*n* = 458)	*p*
Demographic				
Female, *n* (%)	395 (41.4)	187 (37.6)	208 (45.4)	0.018
Age, y(median [IQR])	66.00 [55.00, 77.00]	64.00 [54.00, 75.00]	69.00 [56.00, 79.00]	< 0.001
BMI, kg/m^2^(median [IQR])	27.21 [23.26, 32.32]	27.87 [24.18, 32.65]	26.67 [22.69, 31.89]	0.012
Diagnosis [underlying cardiac causes], *n* (%)	74 (7.7)	55 (11.1)	19 (4.1)	< 0.001
Admission location [Emergency], *n* (%)	541 (56.6)	270 (54.3)	271 (59.2)	0.149
Comorbidities				
aCCI, median [IQR]	6.00 [4.00, 9.00]	6.00 [4.00, 8.00]	7.00 [5.00, 9.00]	< 0.001
Age_score, median [IQR]	3.00 [2.00, 4.00]	3.00 [2.00, 4.00]	4.00 [2.00, 4.00]	< 0.001
Myocardial_infarct, *n* (%)	264 (27.6)	151 (30.4)	113 (24.7)	0.058
Congestive_heart_failure, *n* (%)	424 (44.4)	234 (47.1)	190 (41.5)	0.094
Peripheral_vascular_disease, *n* (%)	137 (14.3)	64 (12.9)	73 (15.9)	0.209
Cerebrovascular_disease, *n* (%)	135 (14.1)	58 (11.7)	77 (16.8)	0.029
Dementia, *n* (%)	38 (4.0)	18 (3.6)	20 (4.4)	0.672
Chronic_pulmonary_disease, *n* (%)	241 (25.2)	129 (26.0)	112 (24.5)	0.646
Rheumatic_disease, *n* (%)	35 (3.7)	16 (3.2)	19 (4.1)	0.554
Peptic_ulcer_disease, *n* (%)	34 (3.6)	21 (4.2)	13 (2.8)	0.327
Mild_liver_disease, *n* (%)	169 (17.7)	78 (15.7)	91 (19.9)	0.109
Diabetes_without_cc, *n* (%)	282 (29.5)	136 (27.4)	146 (31.9)	0.145
Diabetes_with_cc, *n* (%)	160 (16.8)	92 (18.5)	68 (14.8)	0.153
Paraplegia, *n* (%)	34 (3.6)	19 (3.8)	15 (3.3)	0.778
Renal_disease, *n* (%)	335 (35.1)	175 (35.2)	160 (34.9)	0.983
Malignant_cancer,* n* (%)	124 (13.0)	45 (9.1)	79 (17.2)	< 0.001
Severe_liver_disease, *n* (%)	52 (5.4)	18 (3.6)	34 (7.4)	0.015
Metastatic_solid_tumor, *n* (%)	52 (5.4)	11 (2.2)	41 (9.0)	< 0.001
AIDS, *n* (%)	7 (0.7)	2 (0.4)	5 (1.1)	0.27
SEPSIS, *n* (%)	660 (69.1)	334 (67.2)	326 (71.2)	0.208
Treatment, *n* (%)				
Defibrillation	33 (3.5)	18 (3.6)	15 (3.3)	0.908
ventilation	624 (65.3)	312 (62.8)	312 (68.1)	0.096
Laboratory indicators, median [IQR]				
NMLR	6.40 [3.34, 12.61]	5.67 [3.08, 10.17]	7.30 [3.64, 15.01]	< 0.001
WBC × 10^^9^/L	9.40 [6.60, 14.60]	8.90 [6.50, 13.80]	10.25 [6.80, 15.57]	0.013
Basophils, × 10^^9^/L	0.03 [0.01, 0.05]	0.03 [0.01, 0.05]	0.02 [0.00, 0.04]	< 0.001
Eosinophils, × 10^^9^/L	0.09 [0.02, 0.22]	0.11 [0.03, 0.24]	0.07 [0.01, 0.18]	< 0.001
Lymphocytes, × 10^^9^/L	1.21 [0.77, 1.84]	1.32 [0.88, 1.91]	1.12 [0.70, 1.74]	0.001
Monocytes, × 10^^9^/L	0.51 [0.35, 0.78]	0.53 [0.37, 0.78]	0.48 [0.32, 0.77]	0.056
Neutrophils, × 10^^9^/L	7.12 [4.48, 11.91]	6.53 [4.21, 10.95]	7.83 [4.72, 12.88]	0.004
pco2, mmHg	43.00 [36.00, 52.00]	44.00 [37.00, 53.00]	42.00 [35.00, 52.00]	0.171
aado2_calc, mmHg	259.50 [171.95, 488.62]	249.50 [167.25, 496.75]	267.38 [174.51, 480.92]	0.495
PaO2/FiO2	178.00 [88.79, 325.00]	200.00 [97.50, 355.00]	164.50 [82.00, 302.25]	0.003
ph	7.32 [7.21, 7.39]	7.33 [7.25, 7.39]	7.29 [7.18, 7.37]	< 0.001
Lactate, mmol/L	2.80 [1.70, 5.10]	2.40 [1.50, 4.30]	3.50 [1.80, 6.50]	< 0.001
Hematocrit, %	32.40 [27.50, 38.30]	33.10 [28.00, 39.30]	31.40 [26.92, 37.70]	0.008
Hemoglobin, g/L	10.50 [8.80, 12.40]	10.80 [9.00, 12.80]	10.00 [8.60, 12.07]	< 0.001
Platelet, × 10^^9^/L	194.00 [142.00, 266.50]	192.00 [147.00, 262.00]	197.00 [130.00, 278.00]	0.806

aCCI, age-adjusted Charlson Comorbidity Index; AIDS, Acquired immunodeficiency syndrome; NMLR, the (neutrophil + monocyte)/lymphocyte ratio; WBC, white blood cell; diabetes_without_cc, diabetes without complications; diabetes_with_cc, diabetes with complications

**Fig. 2:**
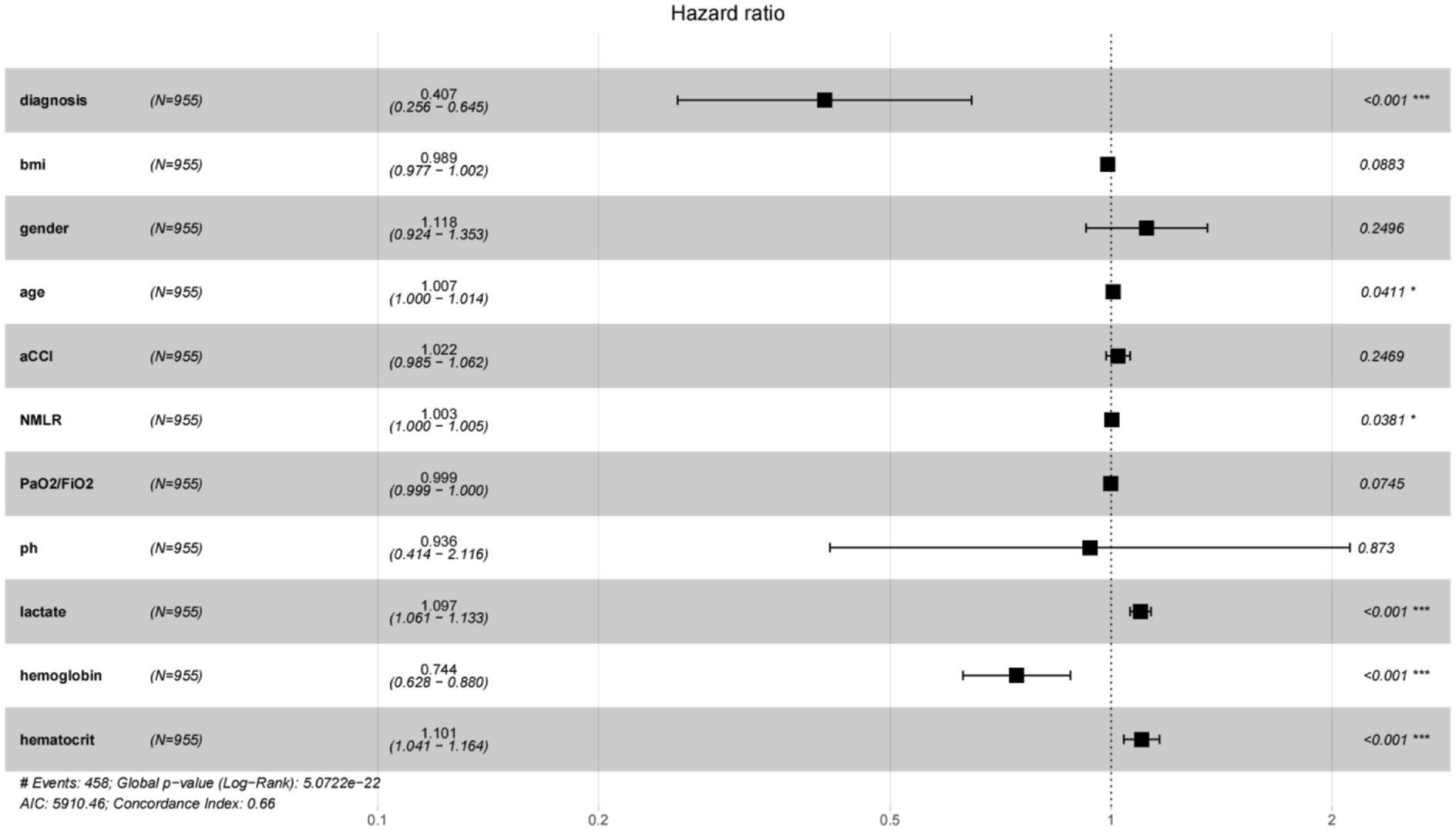
Forest plot shows the variables associated with mortality outcomes after multivariable COX regression. The forest plot shows the variables associated with mortality outcomes after multivariable COX regression. aCCI, age-adjusted Charlson Comorbidity Index; Diagnosis represents an underlying cardiac cause; NMLR, the (neutrophil + monocyte)/lymphocyte ratio

**Fig. 3:**
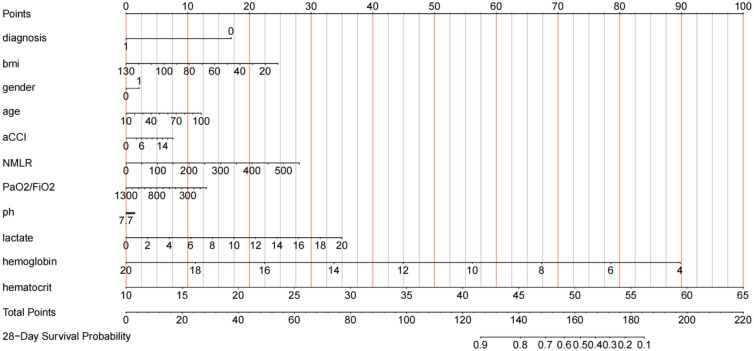
Nomogram based on the results of multivariate Cox regression proportional hazard model. The line segment corresponding to each variable is marked with a scale representing the range of values available for that variable, while the length of the line segment reflects the size of that factor's contribution to the ending event. The total score is obtained by making a line perpendicular to the point count axis at the position of the corresponding variable, and the individual scores for all variables are added together to give the total score. The bottom of the graph represents the corresponding 28-day survival rate. aCCI, age-adjusted Charlson Comorbidity Index; Diagnosis represents an underlying cardiac cause; NMLR, the (neutrophil + monocyte)/lymphocyte ratio

The variables that were statistically different in the univariate analysis above were included in a multivariable Cox proportional hazards regression model. Age (RR 1.007, *p* = 0.0411), NMLR level (RR 1.003, *p* = 0.0381), lactate (RR 1.097, *p* < 0.001), and hematocrit (RR = 1.101, *p* < 0.001) were found to be independent risk factors for patient death after cardiopulmonary resuscitation. The presence of an underlying cardiac cause (RR 0.407, *P* < 0.001), hemoglobin (RR 0.744, *P* < 0.001) was a protective factor (Fig. 2). Based on the results of multivariate COX regression, a nomogram was generated by weighting the score for each factor associated with death after CPR (Fig. 3).

The KM curves determined that the optimal cutoff value of NMLR was 14.2 (Additional file 1). Based on the NMLR cutoff value, patients were divided into a high NMLR group (> 14.2) and a low NMLR group (≤ 14.2). There were 210 patients in the high NMLR group and 745 patients in the low NMLR group. The baseline data of patients in both groups are shown in Table 2. There were no statistically significant differences in gender, age, BMI, etiology, route of admission, CCI score, or treatment regimen. Sepsis was more common in the high NMLR group compared to the low NMLR group (*p* = 0.001), with higher absolute values of leukocytes (*p* < 0.001), monocytes (*p* = 0.001), neutrophils (*p* < 0.001), lactate (*p* = 0.006) and platelets (*p* = 0.002); Eosinophils (*p* < 0.001), basophils (*p* < 0.001), lymphocytes (*p* < 0.001), and ph (*p* = 0.001) were even lower. In terms of clinical outcomes, the high NMLR group had higher in-hospital mortality (*p* < 0.001), lower 28-day survival rate (*p* < 0.001) and more total vasoactive drugs used in the ICU stay. In contrast, length of hospital stay was not statistically different between the two groups of patients.

**Table 2:** Baseline characteristics before and after propensity matching

Before PSM				
	Overall (*n* = 955)	High NMLR (*n* = 210)	Low NMLR (*n* = 745)	*p*
Demographic				
Female, *n* (%)	395 (41.4)	91 (43.3)	304 (40.8)	0.563
age, y (median [IQR])	66.00 [55.00, 77.00]	68.50 [56.00, 79.00]	65.00 [55.00, 76.00]	0.191
BMI, kg/m^2^ (median [IQR])	27.21 [23.26, 32.32]	26.25 [22.75, 31.42]	27.66 [23.50, 32.44]	0.118
Diagnosis [underlying cardiac causes], *n* (%)	74 (7.7)	15 (7.1)	59 (7.9)	0.821
Admission location [Emergency], *n* (%)	541 (56.6)	115 (54.8)	426 (57.2)	0.585
Comorbidities				
aCCI, median [IQR]	6.00 [4.00, 9.00]	6.00 [4.00, 9.00]	6.00 [4.00, 9.00]	0.785
Age_score, median [IQR]	3.00 [2.00, 4.00]	3.50 [2.00, 4.00]	3.00 [2.00, 4.00]	0.358
Myocardial_infarct, *n* (%)	264 (27.6)	63 (30.0)	201 (27.0)	0.437
Congestive_heart_failure, *n* (%)	424 (44.4)	91 (43.3)	333 (44.7)	0.785
Peripheral_vascular_disease, *n* (%)	137 (14.3)	37 (17.6)	100 (13.4)	0.155
Cerebrovascular_disease, *n* (%)	135 (14.1)	26 (12.4)	109 (14.6)	0.475
Dementia, *n* (%)	38 (4.0)	9 (4.3)	29 (3.9)	0.954
Chronic_pulmonary_disease, *n* (%)	241 (25.2)	55 (26.2)	186 (25.0)	0.787
Rheumatic_disease, *n* (%)	35 (3.7)	5 (2.4)	30 (4.0)	0.361
Peptic_ulcer_disease, *n* (%)	34 (3.6)	6 (2.9)	28 (3.8)	0.681
Mild_liver_disease, *n* (%)	169 (17.7)	32 (15.2)	137 (18.4)	0.34
Diabetes_without_cc, *n* (%)	282 (29.5)	56 (26.7)	226 (30.3)	0.345
Diabetes_with_cc, *n* (%)	160 (16.8)	29 (13.8)	131 (17.6)	0.234
Paraplegia, *n* (%)	34 (3.6)	10 (4.8)	24 (3.2)	0.394
Renal_disease, *n* (%)	335 (35.1)	72 (34.3)	263 (35.3)	0.849
Malignant_cancer, *n* (%)	124 (13.0)	22 (10.5)	102 (13.7)	0.268
Severe_liver_disease, *n* (%)	52 (5.4)	8 (3.8)	44 (5.9)	0.312
Metastatic_solid_tumor, *n* (%)	52 (5.4)	11 (5.2)	41 (5.5)	1
AIDS, N (%)	7 (0.7)	1 (0.5)	6 (0.8)	1
Sepsis, *n* (%)	660 (69.1)	166 (79.0)	494 (66.3)	0.001
Treatment, *n* (%)				
Defibrillation	33 (3.5)	7 (3.3)	26 (3.5)	1
Ventilation	624 (65.3)	141 (67.1)	483 (64.8)	0.59
Laboratory indicators, median [IQR]				
WBC, × 10^^9^/L	9.40 [6.60, 14.60]	15.95 [11.53, 20.98]	8.50 [6.10, 12.00]	< 0.001
Basophils, × 10^^9^/L	0.03 [0.01, 0.05]	0.02 [0.00, 0.04]	0.03 [0.01, 0.05]	< 0.001
Eosinophils, × 10^^9^/L	0.09 [0.02, 0.22]	0.02 [0.00, 0.07]	0.12 [0.04, 0.25]	< 0.001
Lymphocytes, × 10^^9^/L	1.21 [0.77, 1.84]	0.62 [0.43, 0.89]	1.40 [1.00, 2.00]	< 0.001
Monocytes, × 10^^9^/L	0.51 [0.35, 0.78]	0.60 [0.35, 1.10]	0.50 [0.35, 0.73]	0.001
Neutrophils, × 10^^9^/L	7.12 [4.48, 11.91]	14.32 [10.22, 19.09]	6.00 [3.98, 9.23]	< 0.001
pco2,mmHg	43.00 [36.00, 52.00]	44.00 [37.00, 54.00]	43.00 [36.00, 52.00]	0.235
aado2_calc,mmHg	259.50 [171.95, 488.62]	290.68 [180.75, 519.09]	252.25 [170.20, 479.00]	0.132
PaO2/FiO2	178.00 [88.79, 325.00]	184.50 [87.39, 288.00]	178.00 [89.00, 330.00]	0.299
ph	7.32 [7.21, 7.39]	7.28 [7.18, 7.36]	7.32 [7.22, 7.39]	0.001
Lactate, mmol/L	2.80 [1.70, 5.10]	3.40 [1.80, 6.10]	2.70 [1.60, 4.80]	0.006
Hematocrit, %	32.40 [27.50, 38.30]	33.25 [28.02, 39.48]	32.10 [27.30, 38.00]	0.065
Hemoglobin, g/L	10.50 [8.80, 12.40]	10.70 [9.10, 12.97]	10.40 [8.70, 12.30]	0.054
Platelet, × 10^^9^/L	194.00 [142.00, 266.50]	211.00 [157.25, 281.00]	189.00 [137.00, 262.00]	0.002
Outcomes				
Death (%)	458 (48.0)	129 (61.4)	329 (44.2)	< 0.001
Los, day (median [IQR])	3.27 [1.61, 7.07]	3.58 [1.73, 7.13]	3.17 [1.54, 7.00]	0.277
In hospital time, day (median [IQR])	9.17 [4.29, 19.08]	7.58 [3.33, 18.95]	9.62 [4.50, 19.12]	0.083
ICU_28,day (median [IQR])	28.00 [4.27, 28.00]	13.94 [2.43, 28.00]	28.00 [4.92, 28.00]	< 0.001
IV_time_sum, hour (median [IQR])	29.00 [9.00, 73.50]	35.00 [12.00, 95.75]	27.00 [9.00, 67.00]	0.073
NE_sum, μg	0.58 [0.00, 17.47]	2.45 [0.00, 28.49]	0.13 [0.00, 15.08]	0.003
Va_sum, IU	0.00 [0.00, 0.00]	0.00 [0.00, 5.42]	0.00 [0.00, 0.00]	0.016
DB_sum, μg	0.00 [0.00, 0.00]	0.00 [0.00, 0.00]	0.00 [0.00, 0.00]	0.674
Sofa score	8.00 [5.00, 12.00]	9.00 [6.00, 11.00]	8.00 [5.00, 12.00]	0.219
GCS score	15.00[14.00, 15.00]	15.00 [14.00, 15.00]	15.00 [14.00, 15.00]	0.698

After PSM				
	Overall (*n* = 382)	High NMLR (*n* = 191)	Low NMLR (*n* = 191)	*p*
Demographic				
Female, *n* (%)	152 (39.8)	80 (41.9)	72 (37.7)	0.464
Age, y (median [IQR])	68.00 [54.00, 78.00]	68.00 [56.00, 78.00]	68.00 [54.00, 76.50]	0.579
BMI, kg/m^2^ (median [IQR])	26.82 [23.08, 32.17]	26.29 [22.89, 31.92]	27.34 [23.72, 32.67]	0.228
Diagnosis [underlying cardiac causes], *n* (%)	33 (8.6)	13 (6.8)	20 (10.5)	0.275
Admission location [Emergency], *n* (%)	215 (56.3)	102 (53.4)	113 (59.2)	0.302
Comorbidities				
aCCI, median [IQR]	7.00 [5.00, 9.00]	6.00 [4.00, 9.00]	7.00 [5.00, 9.00]	0.363
Age_score, median [IQR]	3.00 [2.00, 4.00]	3.00 [2.00, 4.00]	3.00 [2.00, 4.00]	0.603
Myocardial_infarct, *n* (%)	111 (29.1)	59 (30.9)	52 (27.2)	0.499
Congestive_heart_failure, *n* (%)	186 (48.7)	85 (44.5)	101 (52.9)	0.125
Peripheral_vascular_disease, *n* (%)	60 (15.7)	33 (17.3)	27 (14.1)	0.482
Cerebrovascular_disease, *n* (%)	48 (12.6)	26 (13.6)	22 (11.5)	0.643
Dementia, *n* (%)	15 (3.9)	8 (4.2)	7 (3.7)	1
Chronic_pulmonary_disease, *n* (%)	107 (28.0)	50 (26.2)	57 (29.8)	0.494
Rheumatic_disease, *n* (%)	15 (3.9)	4 (2.1)	11 (5.8)	0.114
Peptic_ulcer_disease, *n* (%)	11 (2.9)	5 (2.6)	6 (3.1)	1
Mild_liver_disease, *n* (%)	62 (16.2)	31 (16.2)	31 (16.2)	1
Diabetes_without_cc, *n* (%)	116 (30.4)	50 (26.2)	66 (34.6)	0.095
Diabetes_with_cc, *n* (%)	58 (15.2)	27 (14.1)	31 (16.2)	0.669
Paraplegia, *n* (%)	13 (3.4)	10 (5.2)	3 (1.6)	0.09
Renal_disease, *n* (%)	143 (37.4)	68 (35.6)	75 (39.3)	0.526
Malignant_cancer, *n* (%)	37 (9.7)	16 (8.4)	21 (11.0)	0.489
Severe_liver_disease, *n* (%)	18 (4.7)	8 (4.2)	10 (5.2)	0.809
Metastatic_solid_tumor, *n* (%)	23 (6.0)	9 (4.7)	14 (7.3)	0.39
AIDS, *n* (%)	3 (0.8)	1 (0.5)	2 (1.0)	1
Sepsis, *n* (%)	297 (77.7)	148 (77.5)	149 (78.0)	1
Treatment, *n* (%)				
Defibrillation	13 (3.4)	6 (3.1)	7 (3.7)	1
Ventilation	253 (66.2)	126 (66.0)	127 (66.5)	1
Laboratory indicators, median [IQR]				
WBC, × 10^^9/^L	12.15 [8.20, 17.05]	15.70 [11.50, 20.20]	9.00 [6.60, 13.10]	< 0.001
Basophils, × 10^^9/^L	0.02 [0.00, 0.05]	0.02 [0.00, 0.04]	0.03 [0.01, 0.05]	< 0.001
Eosinophils, × 10^^9/^L	0.05 [0.00, 0.18]	0.02 [0.00, 0.07]	0.14 [0.05, 0.30]	< 0.001
Lymphocytes, × 10^^9/^L	0.93 [0.60, 1.48]	0.62 [0.42, 0.88]	1.40 [1.04, 1.98]	< 0.001
Monocytes, × 10^^9/^L	0.54 [0.35, 0.87]	0.58 [0.33, 1.07]	0.52 [0.36, 0.76]	0.081
Neutrophils, × 10^^9/^L	10.13 [6.10, 15.34]	14.01 [10.25, 18.49]	6.68 [4.56, 9.84]	< 0.001
pco2, mmHg	43.00 [36.00, 53.00]	43.00 [36.00, 52.50]	43.00 [36.00, 53.00]	0.905
aado2_calc, mmHg	266.75 [175.81, 501.38]	276.50 [180.75, 514.25]	260.75 [175.22, 492.50]	0.455
PaO2/FiO2	190.71 [88.57, 314.75]	185.00 [87.00, 288.00]	196.00 [92.00, 326.50]	0.282
Ph	7.30 [7.20, 7.37]	7.29 [7.19, 7.36]	7.32 [7.20, 7.37]	0.282
Lactate, mmol/L	2.90 [1.80, 5.40]	3.20 [1.70, 5.70]	2.80 [1.80, 4.90]	0.799
Hematocrit, %	33.25 [27.83, 39.30]	33.60 [28.10, 39.70]	32.90 [27.35, 38.40]	0.247
Hemoglobin, g/L	10.70 [8.93, 12.80]	10.80 [9.10, 13.15]	10.50 [8.75, 12.40]	0.144
Platelet, × 10^^9/^L	203.00 [152.25, 268.75]	204.00 [153.50, 268.50]	200.00 [147.50, 269.50]	0.967
Outcomes				
Death (%)	196 (51.3)	115 (60.2)	81 (42.4)	0.001
Los, day (median [IQR])	3.75 [1.82, 7.60]	3.56 [1.72, 7.11]	3.97 [1.95, 7.76]	0.521
In hospital time, day (median [IQR])	9.77 [4.47, 20.20]	7.42 [3.12, 18.79]	11.25 [5.42, 21.33]	0.005
ICU_28, day (median [IQR])	28.00 [3.74, 28.00]	13.96 [2.47, 28.00]	28.00 [4.98, 28.00]	0.001
IV_time_sum, hour (median [IQR])	35.00 [11.25, 79.50]	35.00 [12.00, 97.50]	35.00 [11.00, 69.50]	0.397
NE_sum, ug	0.91 [0.00, 18.98]	1.89 [0.00, 26.19]	0.04 [0.00, 14.09]	0.053
Va_sum, IU	0.00 [0.00, 0.00]	0.00 [0.00, 3.30]	0.00 [0.00, 0.00]	0.068
DB_sum, ug	0.00 [0.00, 0.00]	0.00 [0.00, 0.00]	0.00 [0.00, 0.00]	0.974
Sofa score	8.00 [5.00, 12.00]	9.00 [6.00, 11.00]	8.00 [5.00, 12.00]	0.285
GCS score	15.00 [14.00, 15.00]	15.00 [14.00, 15.00]	15.00 [14.00, 15.00]	0.642

aCCI: age-adjusted Charlson Comorbidity Index; AIDS: acquired immunodeficiency syndrome; NMLR: the (neutrophil + monocyte)/lymphocyte ratio; WBC: white blood cell; diabetes_without_cc: diabetes without complications; diabetes_with_cc: diabetes with complications; los: length of ICU stay; IV_time_sum: duration of intravenous dosing; NE_sum: total norepinephrine dosing; Va_sum: total vasopressin dosing; DB_sum: total dopamine dosing; Sofa score: sequential organ failure assessment score; GCS score: Glasgow Coma Scale

**Fig. 4:**
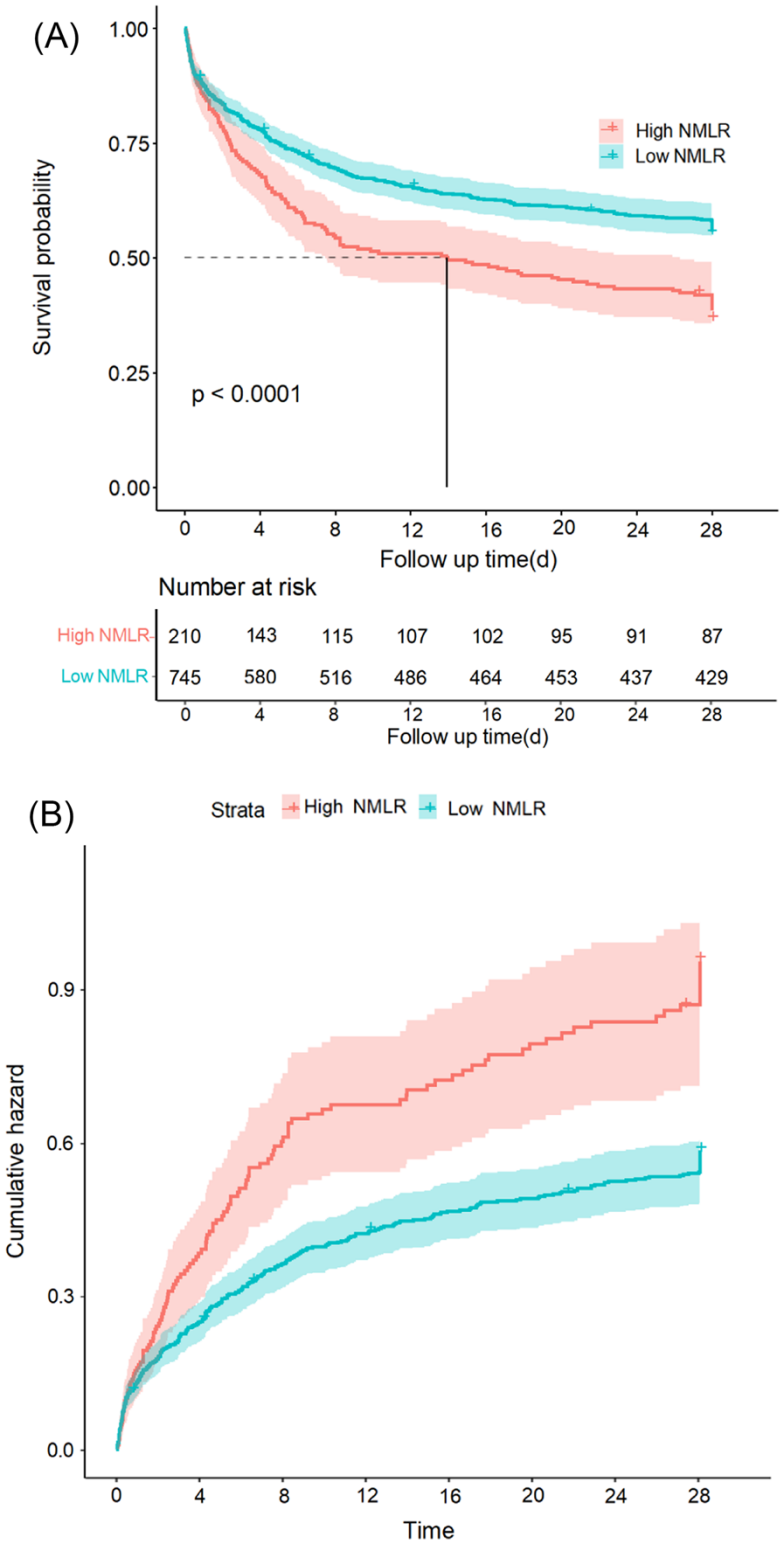
Survival from admission to 28 days of follow-up. Survival from admission to 28 days of follow-up was compared between the High NMLR and Low NMLR groups by log-rank test, **A** Kaplan–Meier survival curves and **B** cumulative risk curves

Using the Kaplan–Meier method, we plotted the KM survival curve and compared the 28-day survival rate of the high NMLR group and the low NMLR group to evaluate the effect of NMLR level on the 28-day survival rate. The cumulative incidence of 28-day death was also plotted. We found that patients in the high NMLR group had a higher mortality rate than those in the low NMLR group at any one time. A log-rank test revealed that *P* < 0.0001, and we can determine that there was a statistical difference in survival between the two groups, with the high NMLR group having a lower 28-day survival rate than the low NMLR group (Fig. 4).

In a univariate analysis based on the grouping of NMLR levels, we found statistically significant differences in ph, lactate, platelet, and the presence of combined sepsis between the two groups (*p* < 0.05) (Table 2).To reduce the effect of confounding factors, we propensity-matched the indicators that differed in the univariate analysis. The results after PSM are shown in Table 2. In addition, SMD values before and after matching are shown in the Additional file 2. There were no statistically significant differences in baseline information between the two groups after propensity matching. In terms of clinical outcomes, the high NMLR group had a higher mortality rate (*p* = 0.001), lower 28-day survival rate (*p* = 0.001) and shorter length of hospital stay (*p* = 0.005) compared to the low NMLR group (Additional file 3).

**Fig. 5:**
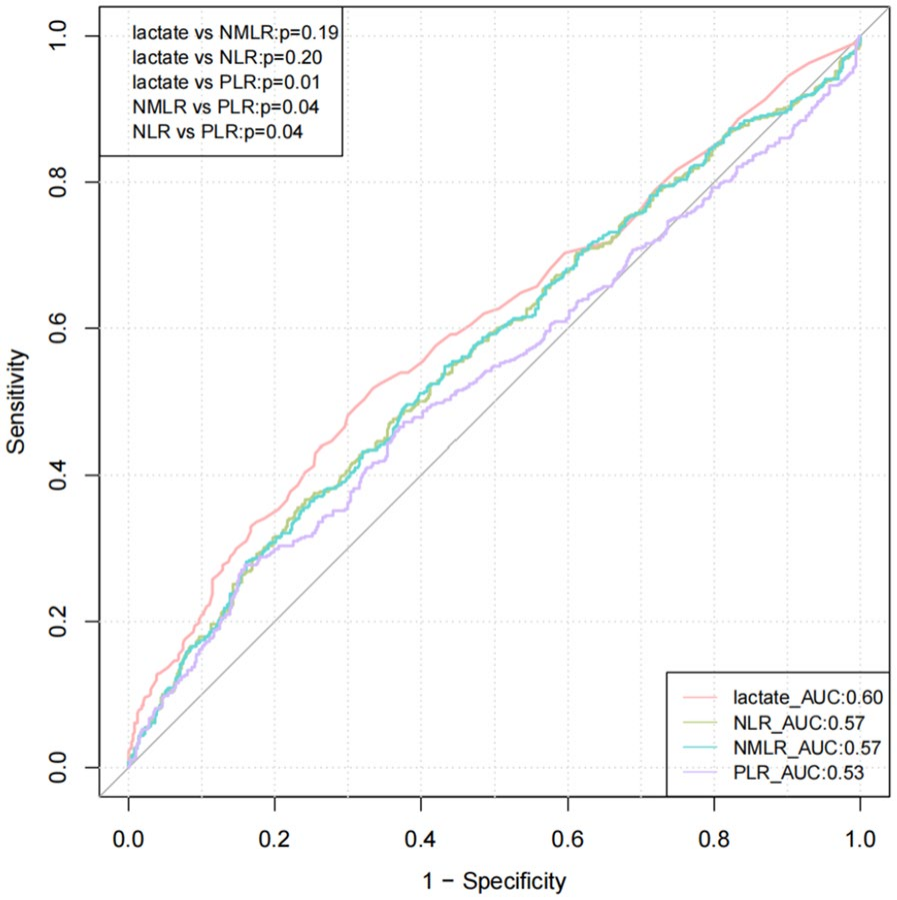
Comparison of ROC curves of NMLR, NLR, PLR and lactate. NLR, neutrophil to lymphocyte ratio; PLR, platelet to lymphocyte rate; NMLR, the (neutrophil + monocyte)/lymphocyte ratio

The baseline values of NMLR, NLR, PLR and lactate in the cohort are shown in Table 3. We compared the NMLR index with NLR, PLR and lactate, and found that the AUC values of lactate, NMLR, NLR and PLR were 0.60, 0.57, 0.57 and 0.53, respectively. However, there was no significant difference among lactate, NMLR and NLR (*p* > 0.05), and the predictive performance of these three indexes was better than PLR. In our data, NMLR and NLR have similar predictive performance (Fig. 5).

**Table 3:** Predictive effect of NMLR and NLR, PLR and lactate levels on mortality after cardiopulmonary resuscitation

	Overall (*n* = 955)	Survival (*n* = 497)	Death (*n* = 458)	*p*
NLR	5.84 [3.02, 11.80]	5.15 [2.80, 9.57]	6.72 [3.31, 14.13]	< 0.001
PLR	156.02 [93.07, 273.16]	149.12 [92.99, 251.23]	163.63 [93.75, 315.33]	0.078
NMLR	6.40 [3.34, 12.61]	5.67 [3.08, 10.17]	7.30 [3.64, 15.01]	< 0.001
lactate	2.80 [1.70, 5.10]	2.40 [1.50, 4.30]	3.50 [1.80, 6.50]	< 0.001

NLR, neutrophil to lymphocyte ratio; PLR, platelet to lymphocyte rate; NMLR, the (neutrophil + monocyte)/lymphocyte ratio

## Discussion

The NMLR, a ratio of the sum of peripheral neutrophil and monocyte counts to lymphocyte counts, is an indicator of combined innate and adaptive immune responses and has shown reliable value in assessing the prognosis of diseases associated with inflammation and immune disorder. As an emerging indicator of inflammation and immune status, elevated NMLR is associated with poor prognosis in diseases, such as acute myocardial infarction and multiple myeloma [8, 9]. However, there are no studies on the correlation between this indicator and the prognosis of patients after cardiopulmonary resuscitation. In this paper, we identified elevated NMLR as a risk factor for poor prognosis in patients after CPR by COX regression analysis, and compared the survival of patients with high and low NMLR levels after CPR by adjusting for confounding factors by propensity score. We found that elevated NMLR levels were associated with increased mortality in patients after cardiopulmonary resuscitation.

Patients with cardiac arrest experience ischemia and hypoxia in all organs of the body, and reperfusion injury occurs after recovery of autonomic circulation by cardiopulmonary resuscitation. Endothelial dysfunction, immune activation and inflammatory response are key events in the process of ischemia reperfusion injury, leading to serious organ dysfunction [10]. During ischaemia–reperfusion injury, leucocytes from the circulating blood are recruited to the site of injury. The main leukocytes involved in this process are neutrophils and, to a lesser extent, monocytes. The recruited neutrophils accumulate at the site of injury and exacerbate local injury by producing locally bioactive amines (histamine and 5-hydroxytryptamine), cytokines (IL-1 and TNF-α) and chemokines, which activate inflammatory signalling pathways in the inflammatory cascade response. This process contributes to increased vascular permeability, leukocyte infiltration and recruitment of circulating monocytes to the site of injury, activating an adaptive immune response and leading to further mediated secondary injury and exacerbated organ dysfunction. Therefore, circulating levels of neutrophils and monocytes help us to assess the inflammatory immune status of the body [11].

Previous study found elevated levels of bone morphogenetic protein (BMP4) in the plasma of patients after cardiopulmonary resuscitation.BMP4 is a potent activator of inflammation in vivo and is involved in tissue inflammation by inducing E-selectin and ICAM-1 to promote the movement, adhesion and extravasation of leukocyte subsets [12]. Therefore, it can be hypothesized that leukocytes are involved in the formation of post-resuscitation syndrome after cardiac arrest, which is associated with inflammatory response, oxidative stress. Changes in leukocyte subpopulations occur during this process and there are different trends in the number of leukocytes in the circulating blood over time. Leukocytes have been shown to be increased in the circulation after cardiac arrest and changes in leukocyte subtype distribution occur. The changes in leukocyte subtypes can be the predictor of the outcome after cardiac arrest [5].

NLR is the ratio of neutrophil count to lymphocyte count, and the correlation between NLR levels and prognosis in patients resuscitated from cardiac arrest has been demonstrated in several previous studies. NLR ≥ 72 at 6 h post-resuscitation is associated with poor neurological prognosis 6 months after resuscitation from cardiac arrest [13]. Bone bridging protein (OPN), a molecule associated with tissue neutrophil infiltration, myeloperoxidase (MPO) and resistin are associated with neutrophil activation and are considered alternative markers of neutrophil recruitment and oxidative burst, and high levels of OPN and MPO at admission in cardiac arrest patients were found to be independent predictors of abnormal EEG changes in the subsequent 48 h in a related study, predicting early risk of secondary neurological injury and was associated with a poor neurological prognosis at 6 months. Thus, neutrophil activation is involved in the pathology of tissue damage after cardiac arrest [14]. In a randomized controlled trial, elevated total white blood cell counts and neutrophil levels measured on the first day in comatose patients resuscitated from out-of-hospital cardiac arrest were found to be significantly associated with 180-day all-cause mortality and an early predictor of poor prognosis [15]. In our study, elevated neutrophil counts in circulating blood were similarly found to be associated with poor prognosis in patients after cardiopulmonary resuscitation.

Monocytes, as members of the innate immune system, are the first line of defense against pathogens and can migrate into and differentiate at sites of injury during acute and chronic inflammation, with a shift in the distribution of monocyte subpopulations involved in the inflammatory response and tissue repair process [16]. There were a randomised trial of a placebo-controlled Tocilizumab intervention in out-of-hospital cardiac arrest patients, a positive correlation was found between the levels of monocytes in the Tocilizumab intervention group and the time to ROSC, Troponin T (TnT), Neurofilament Light Chain (NFL), neuron-specific enolase (NSE), Sequential Organ Assessment (SOFA) score, and Vasoactive–Inotropic Score (VIS).In the peripheral blood of patients after cardiopulmonary resuscitation, monocytes, a subtype of leukocytes, were found to have the same tendency to change over time as neutrophils. Therefore, the levels of monocytes can help us to assess the myocardial and brain injury in patients after cardiac arrest, and also to monitor them dynamically. In the study of Meyer MAS et al., neutrophils and lymphocytes began to decline within 24 h after cardiopulmonary resuscitation, while monocytes showed a significant decline only 72 h after cardiopulmonary resuscitation [5]. Therefore, this may be the reason why there is no significant difference between the prediction effect of NMLR and NLR in this study. However, since monocytes are involved in the damage repair process of acute and chronic inflammation, NMLR may show advantages in continuous monitoring compared with NLR.

In an experimental animal model of acute myocardial infarction, T and B lymphocytes were found to infiltrate into the damaged myocardium. There was an increase in lymphocytes within the infarct zone; however, there was a decrease in circulating lymphocytes. To some extent, the decrease in circulating lymphocytes can reflect the inflammatory situation at the site of injury [8, 9]. Low lymphocyte counts have been found in previous studies to be an independent risk factor for death in even cardiac arrest patients, and it has also been found that prevention of lymphocytopenia may significantly improve the prognosis of critically ill patients [17]. In our study, lymphocyte counts were found to be lower in the death group than in the survival group.

As defined in previous studies, the NMLR is a combination of neutrophil, monocyte and lymphocyte counts and is more comprehensive than the NLR [8, 9]. Compared to NLR, NMLR can better represent the balance between damage and repair. In our study we found that an NMLR > 14.2 was associated with a higher risk of death. The current results suggest that there is immune dysregulation in patients after cardiopulmonary resuscitation, and the NMLR indicator may help us identify patients who need immunomodulatory therapy, which may be followed by attempts to improve clinical outcomes with interventions, such as corticosteroids.

### Limitations

There are several limitations in this study. First, due to the lack of some important information in the public database, including the main cause of the first admission of patients with IHCA, the specific time of cardiac arrest in patients, the duration of resuscitation, the specific time to return of spontaneous circulation, the level of NMLR before cardiac arrest and the use of therapeutic drugs before admission to the ICU, there may be a certain selection bias. Second, the survival rate after CPR in our study was higher than 5–40% survival rate in previous studies, due to our inability to capture the main causes and types of cardiac arrest (shockable, rhythm or asystole), so it may have led to bias. Finally, there was a certain degree of missing data in the included sample, and although we excluded data with > 25% missing amount and filled in the missing values by multiple interpolation, it may still lead to some bias in the results of statistical analysis.

## Conclusion

A multivariate Cox regression proportional hazard model identified age, NMLR level, lactate level and haematocrit size as independent risk factors for patients after CPR. NMLR > 14.2 was associated with higher mortality and was a potential predictor of clinical outcome in patients after CPR.

### Supplementary Information

**Additional file 1:** Distribution of NMLR and the cutoff values.

**Additional file 2:** SMD before and After PSM.

**Additional file 3:** Baseline characteristics of patients with and without recurrent ventricular fibrillation after cardiopulmonary resuscitation.

## Data Availability

We passed the ethics examination of the collaborative institutional training initiative (CITI) and were granted permission to access research data from the MIMIC database.
