# Supplementary material for: The relationship between the level of NMLR on admission and the prognosis of patients after cardiopulmonary resuscitation: a retrospective observational study
Source: Eur J Med Res. 2023 Oct 11;28:424. doi: 10.1186/s40001-023-01407-w (PMC10565961; doi:10.1186/s40001-023-01407-w)
Supplement: Supplementary file 1 — Additional file 1: Distribution of NMLR and the cutoff values. [file 40001_2023_1407_MOESM1_ESM.docx]

Additional file 1.Distribution of NMLR and the cutoff values

| Distribution | | | |
| --- | --- | --- | --- |
| Density | 100  75  50  25  0 | 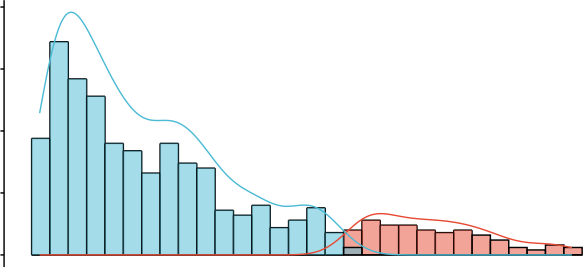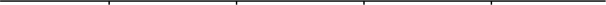  5 10 15 20 | |
| Standardized Log−Rank Statistic | 4  3  2  1  0 | Maximally Selected Rank Statistics  **●**  **●**  **●● ●●**  **●**  **● ●●●**  **● ●**  **●● ●●**  **●● ●**  **● ● ● ● ●**  **●**  **●●**  **● ● ● ● ● ●● ●**  **● ●●●**  **● ● ● ●●●**  **●●**  **● ●● ● ●●**  **● ●●●●**  **● ●**  **●●●**  **● ● ●**  **● ● ● ●**  **● ●● ●**  **● ●●**  **●**  **●**  **●**  **● ● ●**  **●**  **●●**  **● ● ● ● ● ●● ●**  **●● ●**  **● ● ●**  **●**  **●**  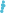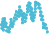 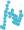**●**  **● ● ● ●**  **● ●**  **●●●**  **● ● ●**  **●● ● ● ●● ●**  **● ●**  **●**  **●**  **●**  **●**  **●● ●**  **●● ●● ● ● ●● ●**  **●**  **●**  **●**  **●● ●**  **● ●**  **●**  **● ● ●**  **● ● ●**  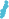 **●** 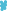**●**  **●**  **●**  **● ●**  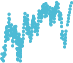  **●**  **●**  **●**  **●**  **●**  **●**  **●**  Cutpoint: 14.23  **●**  **●**  **●**  **●**  **●**  **●**  **●**  **●**  5 10 15 20 | group  **●** High NMLR  **●** Low NMLR |
| NMLR | | | |
|  | | | |

The figure shows the distribution of NMLR and the cutoff values of NMLR, which are divided into the high NMLR group, represented in red, and the low NMLR group, represented in blue, according to the cutoff values.
