# Supplementary material for: The relationship between the level of NMLR on admission and the prognosis of patients after cardiopulmonary resuscitation: a retrospective observational study
Source: Eur J Med Res. 2023 Oct 11;28:424. doi: 10.1186/s40001-023-01407-w (PMC10565961; doi:10.1186/s40001-023-01407-w)
Supplement: Supplementary file 2 — Additional file 2: SMD before and After PSM. [file 40001_2023_1407_MOESM2_ESM.docx]

Additional file 2. SMD before and After PSM

distance

sepsis

platelet

lactate

ph

|  | ● |  | ● | |
| --- | --- | --- | --- | --- |
| ● | ●  ●  ●  ● |  | ●  ● | ● |

0.4

−0.2

0.0

0.2

Standardized Mean Differences

Sample

~~●~~  Unadjusted  ~~●~~  Adjusted
