# Supplementary material for: The relationship between the level of NMLR on admission and the prognosis of patients after cardiopulmonary resuscitation: a retrospective observational study
Source: Eur J Med Res. 2023 Oct 11;28:424. doi: 10.1186/s40001-023-01407-w (PMC10565961; doi:10.1186/s40001-023-01407-w)
Supplement: Supplementary file 3 — Additional file 3: Baseline characteristics of patients with and without recurrent ventricular fibrillation after cardiopulmonary resuscitation. [file 40001_2023_1407_MOESM3_ESM.docx]

Additional file 3. Baseline characteristics of patients with and without recurrent ventricular fibrillation after cardiopulmonary resuscitation

|  | Overall（n=955） | Defibrillation | |  |
| --- | --- | --- | --- | --- |
|  |  | NO  (n=922) | YES  (n=33) | P |
| **Demographic** |  |  |  |  |
| Female,n(%) | 395 (41.4) | 382 (41.4) | 13 ( 39.4) | 0.957 |
| Age,y(median [IQR]) | 66.00[55.00, 77.00] | 64.32 (16.25) | 70.64 (11.83) | 0.027 |
| Bmi,kg/m^2^(median [IQR]) | 27.21[23.26, 32.32] | 28.66 (8.22) | 29.17 (5.87) | 0.727 |
| diagnosis [underlying cardiac causes],n(%) | 74 (7.7) | 69 ( 7.5) | 5 ( 15.2) | 0.198 |
| admissionlocation[Emergency],n(%) | 541 (56.6) | 526 (57.0) | 15 ( 45.5) | 0.253 |
| **Comorbidities** |  |  |  |  |
| aCCI(median [IQR]) | 6.00[4.00, 9.00] | 6.00[4.00, 9.00] | 7.00[5.00, 9.00] | 0.610 |
| age_score(median [IQR]) | 3.00[2.00, 4.00] | 3.00[2.00, 4.00] | 4.00[3.00, 4.00] | 0.129 |
| myocardial_infarct,n(%) | 264 (27.6) | 249 (27.0) | 15 (45.5) | 0.033 |
| congestive_heart_failure,n(%) | 424 (44.4) | 403 (43.7) | 21 (63.6) | 0.037 |
| peripheral_vascular_disease,n(%) | 137 (14.3) | 131 (14.2) | 6 (18.2) | 0.699 |
| cerebrovascular_disease,n(%) | 135 (14.1) | 127 (13.8) | 8 (24.2) | 0.149 |
| dementia,n(%) | 38 (4.0) | 35 (3.8) | 3 (9.1) | 0.282 |
| chronic_pulmonary_disease,n(%) | 241 (25.2) | 241 (25.2) | 235 (25.5) | 0.456 |
| rheumatic_disease,n(%) | 35 (3.7) | 34 (3.7) | 1 (3.0) | 1.000 |
| peptic_ulcer_disease,n(%) | 34 (3.6) | 30 (3.3) | 4 (12.1) | 0.026 |
| mild_liver_disease,n(%) | 169 (17.7) | 163 (17.7) | 6 (18.2) | 1.000 |
| diabetes_without_cc,n(%) | 282 (29.5) | 273 (29.6) | 9 (27.3) | 0.924 |
| diabetes_with_cc,n(%) | 160 (16.8) | 157 (17.0) | 3 (9.1) | 0.336 |
| paraplegia,n(%) | 34 (3.6) | 33 (3.6) | 1 (3.0) | 1.000 |
| renal_disease,n(%) | 335 (35.1) | 324 (35.1) | 11 (33.3) | 0.978 |
| malignant_cancer,n(%) | 124 (13.0) | 123 (13.3) | 1 (3.0) | 0.142 |
| severe_liver_disease,n(%) | 52 (5.4) | 52 (5.6) | 0 (0.0) | 0.311 |
| metastatic_solid_tumor,n(%) | 52 (5.4) | 51 (5.5) | 1 (3.0) | 0.817 |
| aids,n(%) | 7 (0.7) | 7 (0.8) | 0 (0.0) | 1.000 |
| sepsis,n(%) | 660 (69.1) | 631 (68.4) | 29 (87.9) | 0.029 |
| **Treatment** |  |  |  |  |
| ventilation,n(%) | 624 (65.3) | 324 (35.1) | 7 (21.2) | 0.143 |
| **Laboratory indicators** |  |  |  |  |
| NMLR(median [IQR]) | 6.40[3.34, 12.61] | 6.41[3.36, 12.61] | 5.26[3.23, 12.14] | 0.773 |
| WBC(median [IQR]) | 9.40[6.60, 14.60] | 9.40[6.70, 14.57] | 9.90[6.50, 16.80] | 0.583 |
| basophils(median [IQR]) | 0.03[0.01, 0.05] | 0.03[0.01, 0.05] | 0.03[0.00, 0.04] | 0.752 |
| eosinophils(median [IQR]) | 0.09[0.02, 0.22] | 0.09[0.02, 0.22] | 0.14[0.00, 0.23] | 0.875 |
| lymphocytes(median [IQR]) | 1.21[0.77, 1.84] | 1.21[0.77, 1.83] | 1.44[0.78, 2.04] | 0.364 |
| monocytes(median [IQR]) | 0.51[0.35, 0.78] | 0.52[0.35, 0.77] | 0.50[0.34, 1.03] | 0.831 |
| neutrophils(median [IQR]) | 7.12[4.48, 11.91] | 7.11[4.49, 11.79] | 7.57[4.18, 13.94] | 0.776 |
| pco2(median [IQR]) | 43.00[36.00, 52.00] | 43.00[36.00, 52.00] | 39.00[36.00, 55.00] | 0.769 |
| aado2_calc(median [IQR]) | 259.50[171.95, 488.62] | 259.50[171.70, 487.42] | 266.75[177.25, 502.25] | 0.840 |
| PaO2/FiO2(median [IQR]) | 178.00[88.79, 325.00] | 178.00[88.57, 325.00] | 191.43[111.00, 397.50] | 0.188 |
| ph(median [IQR]) | 7.32[7.21, 7.39] | 7.32[7.21, 7.39] | 7.27[7.20, 7.38] | 0.432 |
| lactate(median [IQR]) | 2.80[1.70, 5.10] | 2.80[1.70, 5.10] | 2.60[1.10, 4.90] | 0.217 |
| hematocrit(median [IQR]) | 32.40[27.50, 38.30] | 32.40[27.50, 38.20] | 32.50[28.30, 38.40] | 0.576 |
| hemoglobin(median [IQR]) | 10.50[8.80, 12.40] | 10.50[8.80, 12.40] | 10.60[8.50, 13.10] | 0.548 |
| platelet(median [IQR]) | 194.00[142.00, 266.50] | 194.00[142.00, 265.75] | 196.00[154.00, 269.00] | 0.601 |
| **Outcomes** |  |  |  |  |
| death (%) | 458 (48.0) | 443 (48.0) | 15 (45.5) | 0.908 |
| Los,day (median [IQR]) | 3.27[1.61, 7.07] | 3.15[1.53, 6.73] | 7.60[5.00, 15.85] | <0.001 |
| In hospital time,day (median [IQR]) | 9.17[4.29, 19.08] | 9.12[4.12, 18.96] | 16.04[6.75, 21.58] | 0.018 |
| ICU_28,day (median [IQR]) | 28.00[4.27, 28.00] | 28.00[4.09, 28.00] | 28.00[7.62, 28.00] | 0.323 |
| IV_time_sum,hour (median [IQR]) | 29.00[9.00, 73.50] | 28.00[9.00, 70.75] | 80.00[23.00, 131.00] | 0.005 |
| NE_sum | 0.58 [0.00, 17.47] | 0.48 [0.00, 17.25] | 3.91 [0.00, 22.68] | 0.235 |
| Va_sum | 0.00 [0.00, 0.00] | 0.00 [0.00, 0.00] | 0.00 [0.00, 25.08] | 0.012 |
| DB_sum | 0.00 [0.00, 0.00] | 0.00 [0.00, 0.00] | 0.00 [0.00, 4.35] | 0.010 |
| Sofa score | 8.00 [5.00, 12.00] | 8.00[5.00, 12.00] | 10.00[8.00, 13.00] | 0.033 |
